# Complete mitochondrial genome of a cavefish *Sinocyclocheilus ronganensis* (Cypriniformes: Cyprinidae)

**DOI:** 10.1080/23802359.2017.1280696

**Published:** 2017-02-23

**Authors:** Fuguang Luo, Jie Huang, Anyou He, Tong Luo, Huanjia Zhou, Yanhong Wen

**Affiliations:** a Liuzhou Aquaculture Technology Extending Station, Liuzhou, China;; b Lab of Fishery and Ecological environment, Guangxi Academy of Fishery Sciences, Nanning, China

**Keywords:** Cavefish, mitochondrial genome, *Sinocyclocheilus ronganensis*

## Abstract

In this study, we report the complete mitochondrial genome of *Sinocyclocheilus ronganensis.* The whole mitochondrial genome is 16,587 bp with an accession number KX778473, and consists of 13 protein-coding genes, 2 ribosomal RNAs, 22 transfer RNAs genes, and a 936 bp control region. Phylogenetic analysis shows that *S. ronganensis* is close to cave-restricted *S. anophthalmus* and surface-dwelling *S. grahami.* The complete mitogenome of *S. ronganensis* may provide useful information for studying the genetic mechanism of cavefish, and enrich the fish mitochondrial genome resource for further research.

*Sinocyclocheilus ronganensis*, a cavefish and is newly found in the karst river of Rong’an county, Southwestern China (Luo et al. 2016). Like other surface-dwelling species (Zhao & Zhang 2009; Meng et al. 2013), *S. ronganensis* has normal eyes, full-cover scales but larger than most of them, and it is a good model for studying the genetic status and evolutionary by comparing the mitochondrial genome.

The *S. ronganensis* sample was collected from subterranean river of Rong’an county (24°59′59″N, 109°28′02″E), Southwestern China in March, 2016, and the specimen was stored in Liuzhou Aquaculture Technology Extending Station, Liuzhou, China. In this study, the complete mtDNA was sequenced using the Illumina Hiseq4000 platform with *de novo* strategy, and then the phylogenetic tree was established using the neighbour-joining (NJ) method.

The complete mitochondrial genome of *S. ronganensis* had been deposited in the GenBank with an accession number KX778473. The mitochondrial genome was 16,587 bp in length, with the base composition of 31.57% A, 25.41% T, 26.82% C, 16.21% G, and an AT bias of 56.97%. Besides, it consisted of 13 protein-coding, 2 ribosomal RNA (rRNA), 22 transfer RNA (tRNA) genes, and 1 control region (D-loop). So far, the arrangement of these genes was identical to that found in the species of *Sinocyclocheilus* (Wu et al. 2010; He et al. 2016; Peng et al. 2017). Except for *ND6* gene and eight tRNA genes, all other genes were located on the heavy strand (H-strand). All the 13 protein-coding genes contained the same start codon ATG except the gene *COXI*, which contained GTG instead, however, the termination codons of the 13 protein-coding genes were different, with either TAA, T––, TA–, or TAG. 22 tRNA genes were ranged from 67 to 76 bp in size, and one 12S rRNA and one 16S rRNA with corresponding sizes of 954 and 1680 bp, respectively. A total of 936 bp control region (D-loop) was located between tRNA-Phe and tRNA-Pro. In all genes, the overlaps and spaces might be existed in adjacent gene, for example, *tRNA-Asn* (32 bp spaces), *tRNA-Asp* (13 bp spaces), *ATP8* (7 bp overlaps), *ND4L* (7 bp overlaps) were found obviously.

*Sinocyclocheilus ronganensis* and *S. grahami* had the same lift-style, and also had the same normal eyes and scales, but interesting, in our phylogenetic tree, the surface-dwelling *S. grahami* and the cave-restricted *S. anophthalmus* first clustered together and later this group clustered with *S.ronganensis* (Figure 1), it suggested that phenotypic convergence might be caused by similar environmental conditions despite the difference in mitochondrial evolution of *Sinocyclocheilus*, and this results of this study was also in agreement with previous reports (Xiao et al. 2005; He et al. 2016; Peng et al. 2017).

**Figure 1.**
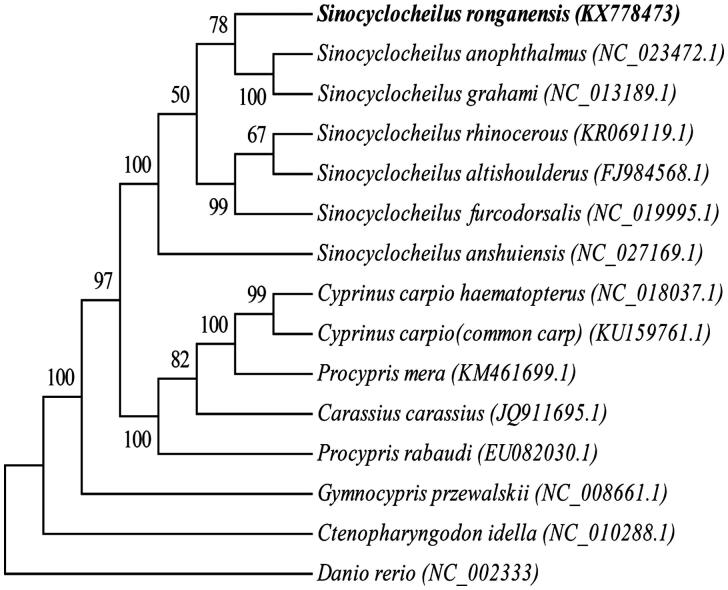
Neighbour-joining phylogenetic tree based on the mitochondrial genome of *S. ronganensis* and other 13 affinis fishes using MEGA6.06, Danio rerio served as an outgroup species.
